# Automating generation of textual class definitions from OWL to English

**DOI:** 10.1186/2041-1480-2-S2-S5

**Published:** 2011-05-17

**Authors:** Robert Stevens, James Malone, Sandra Williams, Richard Power, Allan Third

**Affiliations:** 1 School of Computer Science, The University of Manchester, Oxford Road, Manchester, M13 9PL, UK; 2 European Bioinformatics Institute, Wellcome Trust Genome Campus, Cambridge, CB10 1SD, UK; 3 Department of Computing, Open University, Walton Hall, Milton Keynes, MK7 6AA, UK

## Abstract

**Background:**

Text definitions for entities within bio-ontologies are a cornerstone of the effort to gain a consensus in understanding and usage of those ontologies. Writing these definitions is, however, a considerable effort and there is often a lag between specification of the main part of an ontology (logical descriptions and definitions of entities) and the development of the text-based definitions. The goal of natural language generation (NLG) from ontologies is to take the logical description of entities and generate fluent natural language. The application described here uses NLG to automatically provide text-based definitions from an ontology that has logical descriptions of its entities, so avoiding the bottleneck of authoring these definitions by hand.

**Results:**

To produce the descriptions, the program collects all the axioms relating to a given entity, groups them according to common structure, realises each group through an English sentence, and assembles the resulting sentences into a paragraph, to form as ‘coherent’ a text as possible without human intervention. Sentence generation is accomplished using a generic grammar based on logical patterns in OWL, together with a lexicon for realising atomic entities. We have tested our output for the Experimental Factor Ontology (EFO) using a simple survey strategy to explore the fluency of the generated text and how well it conveys the underlying axiomatisation. Two rounds of survey and improvement show that overall the generated English definitions are found to convey the intended meaning of the axiomatisation in a satisfactory manner. The surveys also suggested that one form of generated English will not be universally liked; that intrusion of too much ‘formal ontology’ was not liked; and that too much explicit exposure of OWL semantics was also not liked.

**Conclusions:**

Our prototype tools can generate reasonable paragraphs of English text that can act as definitions. The definitions were found acceptable by our survey and, as a result, the developers of EFO are sufficiently satisfied with the output that the generated definitions have been incorporated into EFO. Whilst not a substitute for hand-written textual definitions, our generated definitions are a useful starting point.

**Availability:**

An on-line version of the NLG text definition tool can be found at http://swat.open.ac.uk/tools/. The questionaire and sample generated text definitions may be found at http://mcs.open.ac.uk/nlg/SWAT/bio-ontologies.html.

## Background

**Figure 1:**
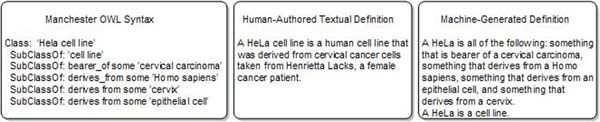
**OWL and natural language definitions for the HeLA cell-line.** This shows an example of the OWL and hand-written textual definition for the HeLa cell line class as seen in EFO. We can see from this that the definitions are similar, in that they both say what a HeLa cell is, but the hand-written one brings in more background information, such as the name of the individual whence the cells came. The rightmost pane shows the definition generated by the version of our program used for the second evaluation study.

This paper presents a prototype tool for generating textual definitions for an ontology from logical definitions using the Experimental Factor Ontology (EFO) [1] as a case study. The heart of ontology building is the definition of entities in a domain. A definition states what kind of thing the described entity is and how it is distinguished from other entities of the same kind. As such, a definition states how an entity can be distinguished or recognised from other entities. Such definitions come in two styles with a common core aim: natural language or text definitions of an entity and logical definitions of an entity. Figure 1 shows an axiomatic description in OWL and a hand-written textual definition for the HeLa cell line from EFO (left and central panes). The information within the two types of definition is similar (they both talk of cells that come from a human cervical carcinoma; the hand-written, however, also gives the information of the individual human whence the cells came), but they differ in style of rendering and apparent ease of reading.

The provision of textual definitions is one of the OBO Foundry [2] criteria; they are a cornerstone of making an ontology usable by its human users. By distinguishing one entity from another, a definition should promote understanding and clarity in its community of users. The definition should diminish ambiguity in annotation of entities—the primary use of ontologies in bioinformatics [3].

While textual definitions are human-facing, logical definitions are primarily machine-facing. They allow ontologies to be built and deployed with the support of automated reasoners [3-6]. The explicit axiomatisation can be checked for its logical coherence (to remove contradictory statements) and to complete the subsumption hierarchy through the inference of subsumption or is-a relationships. As part of a software application, this axiomatisation can also be used to dynamically query the ontology. For example, common queries in the Gene Expression Atlas [7] include searches for genes from a particular organism or organisms, for particular types of disease (e.g. all cancers) and for particular tissue (e.g. all cell lines derived from breast).

Our target ontology, the Experimental Factor Ontology (EFO), is an application ontology used to describe experimental variables in functional genomics data [1]. EFO uses the Web Ontology Language (OWL) [8] to produce a rich, axiomatic description of classes in the domain. The Gene Expression Atlas at EBI has successfully applied EFO in exactly this manner; curated data are annotated with ontology terms and the axiomatisation is used to drive querying [7]. Through descriptions of the entities within a domain and the relationships between those entities, EFO meets its aims through a standard application of ontologies to describing and querying data [3]. Part of the application of ontologies in this way is to present the ontology in question in as comprehensible a manner as possible.

EFO has many logical definitions, but at the start of this work had few manually written text definitions. This is because the primary foci of EFO were three-fold: i) to provide coverage for functional genomics data by importing reference ontology classes or creating new classes where suitable reference classes did not exist, ii) to add user-friendly labels and synonyms to these classes to aid in searching and understanding for a wide diversity of users, iii) to create axiom-rich class descriptions in OWL. The development of EFO concentrated primarly on the logical description of its entities because these were used in order to power querying and browsing in the initial application requirements. This allowed the ontology hierarchies displayed in the interface to be easily manipulated and changed, simply by adding additional defined classes, making them more maintainable and easier to adapt to user requirements that could potentially evolve over time. This modular approach to ontology design is similar to that described in [6]. As a consequence of this prioritization, natural language descriptions of many of the classes in EFO are absent. We expect this situation where natural language definitions lag behind logical definitions to be prevalent in many ontologies that use this approach.

Although capturing definitions in OWL statements is powerful, such formal language can be confusing to a user not familiar with OWL [9]. EFO, along with other ontologies, needs both the logical and textual definitions of entities; one for human users and one for the machine to use to help users meet their goals; thus there is a need for both forms of description within EFO and other ontologies. As a result we would like both logical and textual definitions for the classes in EFO; authoring both is time consuming and there is an issue of keeping them consistent with each other over time. A system that can automatically generate satisfactory (if not ideal) text definitions (see right pane of Figure 1) from the logical descriptions brings obvious advantages, since maintenance of both types of definition is effectively devolved to curating only the logical description.

### Generating texts From ontologies

The correlation of an axiom in OWL and a natural language sentence is intuitive, and generating natural language statements from ontologies is a widespread approach. The task of generating texts from ontologies has been called ‘ontology verbalisation’ (see [10]). A major application of ontology verbalisation has been controlled natural languages (CNL) as a means of both reading and authoring ontologies, the latter avoiding the ambiguities of computational processing of uncontrolled natural language, whilst having the appeal of a ‘natural’ feel to the language. Attempto Controlled English (ACE) [11], Processable English (PENG) [12], and Controlled Language for Ontology Editing (CLOnE) [13], are all examples of CNLs that have been used in ontology verbalisation. Ontology authoring tools such as What You See Is What You Meant (WYSIWYM) [14] and ROO [15,16] allow ontology authors to use highly specified natural language correlates of ontology constructs, coupled with lexicalisations of an ontology’s entities, to create axioms through natural language sentences, though these two do not *per se* act as verbalisers of OWL.

**Figure 2:**
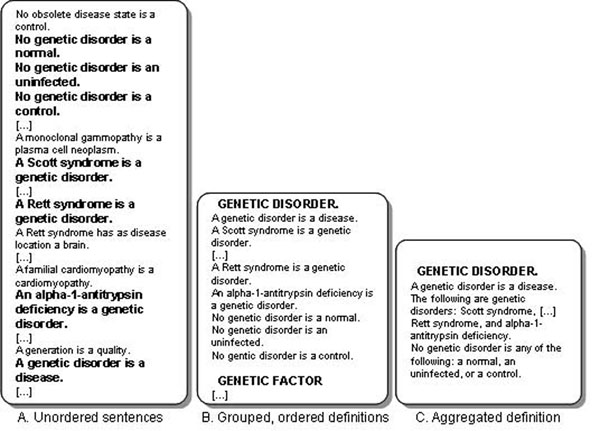
**Ungrouped, grouped, and grouped and aggregated verbalisations of OWL descriptions**. The left-hand box (A) shows a list of ungrouped sentences, each representing an axiom from EFO. The sentences appear in the order in which they occur in the input file. The middle box (B) shows the emboldened sentences from (a) sentences *grouped* according to the ‘subject’ or topic of the sentence. In this case, a genetic disorder. This gathers all the sentences pertinent to genetic disorder into one place. The right-hand box (C) shows the *aggregated* version of the grouped output. The repetition of the subclass axioms is replaced by a list construct.

These systems can act as verbalisers for OWL as well as a means of authoring, and have varying aims and limitations: for instance, some are concerned only with ABox (instances or individuals in OWL) verbalisation (e.g., [17,18]); others produce only separate sentences, one for each OWL axiom (e.g., [19]) (see the supplementary information for an example from EFO generated by ACEView [20]; this shows each axiom from EFO as a separate sentence appearing in the order that the axiom concerned is found in the file). Figure 2 (panel A) shows a similar unordered, sentence per axiom output from EFO. Table 1 gives a comparison of the systems that verbalise OWL.

**Table 1:** Comparisons of OWL verbalisers

System	Tbox	Abox	Coverage	Grouping	Aggregation	Lexicon	Domain
ACE [19]	Yes	Yes	OWL-2	Yes	No	Automatic	Generic
ROA [35]	Yes	Yes	Unclear	Unclear	Yes	Automatic	Generic
SWOOP [23]	Yes	Unclear	OWL-DL	No	No	Automatic	Generic
MIAKT [36]	No	Yes	RDF	Yes	Yes	Handcrafted	Specific
NaturalOWL [18]	Yes	Yes	OWL-DL	Yes	Yes	User-defined	Specific
GINO [37]	Yes	Unclear	Unclear	No	No	Automatic	Generic
LIBER [17]	No	Yes	RDF	Yes	Yes	User-defined	Specific
SWAT Tools	Yes	Yes	OWL-2	Yes	Yes	Automatic	Generic

Comparison of OWL verbalisers. ‘TBox’ contains the ontology’s classes and properties; ‘ABox’ contains the individuals and the assertions upon them. Coverage is approximate: for instance, ACE and SWAT cover nearly all of OWL-2, but with a few omissions. ‘Lexicon’ indicates the source of lexical entries for atomic entities; ‘Domain’ is generic if the system can produce text for any ontology (of the stated OWL coverage) with English-based names, and specific if handcrafted lexical entries or grammar rules are needed.

Ontology authoring tools such as Protégé [21], topBraid Composer [22], SWOOP [23] and Neon Toolkit [24] take a different approach. Ontologies in OWL are collections of axioms, but these tools take a ‘frame’ based view, grouping axioms on a topic such as a class or individual together for easier comprehension by users; such a frame view is not part of OWL, but is a typical presentation mechanism. It is accepted within psycholinguistics that unordered collections of sentences are difficult to comprehend [25-27]; full comprehension of a text depends on inferences by the reader, and the more the text guides such inferences through appropriate organisation, the easier the comprehension task becomes. Organisation is achieved partly through structural units such as paragraphs (the standard one-idea-per-paragraph is a natural correlate of the concept in an ontology) and partly through ‘discourse markers’ — the linking phrases that indicate relationships between portions of text. Another potential barrier to comprehension is multiple repetition of the same sentence form: Figure 2 illustrates this phenomenon in EFO, showing a progression from unordered sentences with one sentence per axiom, through grouped sentences where axioms pertinent to classes are gathered, and finally to grouped sentences with those sentences with relationships in common are aggregated.

Our work addresses these issues by using some standard techniques from computational linguistics and applying them to OWL verbalisation to make the verbalised text more readable, rather than realising axioms one by one. We apply rules for grouping and aggregation [28], using generic methods applicable to any ontology, so as to provide coherent descriptions for each class (or individual or property). As Table 1 indicates, our approach (Semantic Web Authoring (SWAT) Tools) uses similar techniques to other verbalisers, but differs by verbalising (nearly) all of OWL 2, for any ontology with English identifiers or labels, producing descriptions for individuals, classes and properties, with increased attention to fluency and appropriate lexicalisation without any user input. In this work, these techniques are applied to the specific ontology verbalisation task of generating natural language ‘definitions’, where the paragraph-based rendering is inherently more appropriate than a sentence-by-sentence verbalisation, since the class or concept is the intuitive correlate of the paragraph and, as Figures 1 and 2 show, such natural language definitions are narrative in structure, rather than collections of sentences.

From a computational linguistics perspective, ontology verbalisation has some unusual features. Most applications in natural language generation aim to produce high-quality text in restricted domains for which specialised text-planners, grammars and lexicons have been developed [29]. In verbalising any ontology we aim for texts that are useful and understandable, but not necessarily of the highest quality, using methods that are domain-general. The challenge is thus to find generic techniques for:

1. grouping related axioms on the same class;

2. realising logical patterns in English;

3. aggregating axioms sharing a common pattern, such as use of the same property, so that they can be expressed efficiently in a single sentence, and

4. inferring lexical entries for atomic entities (classes and properties) from identifiers and labels in the ontology, with due attention to details like correct parts of speech and plural forms.

These aims are orientated to the coherency of the generated language. For a text definition we need to *group together* related axioms that relate to the concept being defined. A typical presentation of OWL, which is a collection of axioms, is as ‘frames’, so that all the axioms for a given ‘subject’ entity are presented together (see Figure 1); we will need some similar grouping mechanism for generating natural language definitions. Such descriptions can have many relationships of the same kind, such as an entity having multiple parts, and these need to be *aggregated* to reduce needless redundancy.

As well as grouping and aggregation, other questions arise in generating textual definitions:

1. How rigorously should the semantics of OWL be preserved? For example, a simple existential restriction in OWL, such as *HeLa derives_from some ’Homo sapiens’,* means that each and every instance of HeLa is derived from at least one Homo sapiens (but may also derive from some other entity as well). What is the balance between preserving OWL’s semantics and having readable English?

2. How much (if any) of the formal ontological nature of the logical definitions should be preserved? EFO uses the Relation Ontology [30] and so we have axioms such as *Homo sapiens bearer_of some cervical_carcinoma;* should we use ‘bearer of’ here, or some other rendering of such ontologically formal properties (or indeed both)?

We present a prototype for generating textual definitions from OWL using EFO as our ‘test-bed’. We have started to explore the appropriateness of our verbalisations for natural language definitions with informal surveys of potential users. The results already look promising and presentation of generated text definitions to users has suggested ways in which our techniques can be improved.

## Results

Results for Survey 1 are summarised in Table 2 An example of verbalisations from this first iteration is shown in Table 3.

**Table 2:** Results from Survey One

Judgements	1	2	3	4	5
Totals	5.9% (11)	9.1% (17)	27.3% (51)	32.1% (60)	25.7% (48)

Summary of survey results on natural language definitions from iteration 1. Question: ‘How understandable are the definitions?’ Judgements range from 1 (not understandable) to 5 (understandable). The survey was completed by 21 people (questions did not require an answer).

**Table 3:** Examples of output used in Survey One

Class label	OWL axioms (Manchester syntax)	Generated Natural Language Definition
22rv1	SubClassOf: ’cell line’ bearer_of some ’prostate carcinoma’ derives_from some ’Homo sapiens’ derives_from some prostate	A 22rv1 is a cell line. A 22rv1 is all of the following: something that is bearer of a prostate carcinoma, something that derives from a homo sapiens, and something that derives from a prostate.

HeLa	SubClassOf: ’cell line’ bearer_of some ’cervical carcinoma’ derives_from some ’Homo sapiens’ derives_from some ’epithelial cell’ derives_from some cervix	A he la is a cell line. A he la is all of the following: something that is bearer of a cervical carcinoma, something that derives from a homo sapiens, something that derives from an epithelial cell, and something that derives from a cervix.

Ara-C-resistant murine leukemia	SubClassOf: ’cell line’ has subclass b117h* has subclass b140h*	A ara c resistant murine leukemia is a cell line. A b117h, and a b140h are kinds of ara c resistant murine leukemias.

GM18507	SubClassOf: ’cell line’ has_quality some male derives_from some ’Homo sapiens’ derives_from some lymphoblast	A gm18507 is a cell line. A gm18507 is all of the following: something that has as quality a male, something that derives from a homo sapiens, and something that derives from a lymphoblast.

BDCM	SubClassOf: ’cell line’	A bdcm is a cell line.

Example of natural language definitions generated from corresponding OWL axioms from the first iteration (Survey 1). *Note: these subclass relations are placed on the subclasses but we illustrate them here for context.

**Figure 3:**
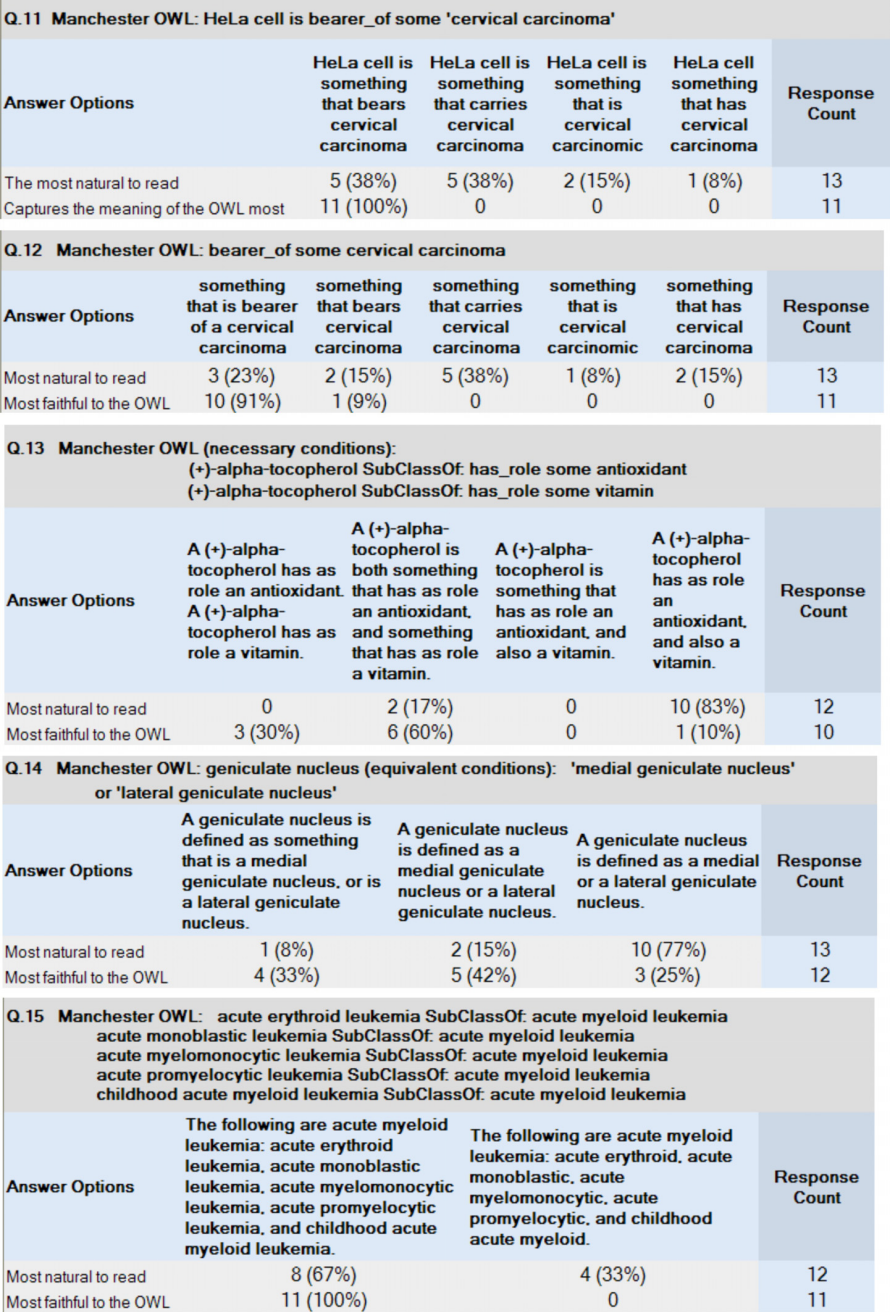
**Alternative renderings for Survey Two**. Alternative renderings for a selection of definitions (Survey 2, Part 2). Participants were asked, in two separate questions, to pick which they thought was the most natural to read and which best captured the meaning of the OWL.

For Survey 2, the results are summarised in Table 4, and examples of verbalisations generated by the program are shown in Table 5. Results for the alternative hand-crafted definitions can be seen in Figure 3.

**Table 4:** Results from Survey Two

Judgements	1	2	3	4	5
Totals	3.6% (5)	5.0% (7)	10.8% (15)	37.4% (52)	43.2% (60)

Summary of results on natural language definitions for the second iteration (Survey 2 Part 1). Question ‘How well does the text capture the OWL meaning?’ Judgements range from 1 (Not at all) to 5 (Totall captured meaning). The survey was completed by 16 people (questions did not require an answer).

**Table 5:** Examples of output Used in Survey Two

Class label	OWL axioms (Manchester syntax)	Generated Natural Language Definition
HeLa	bearer_of some ’cervical carcinoma’ derives_from some ’Homo sapiens’ derives_from some ’epithelial cell’ derives_from some cervix SubClassOf: ’cell line’	A HeLa is all of the following: something that is bearer of a cervical carcinoma, something that derives from a Homo sapiens, something that derives from an epithelial cell, and something that derives from a cervix. A HeLa is a cell line.

4470	derives_from some ’Mus musculus’ derives_from some ’bone marrow’ SubClassOf: ’cell line’	A 4470 is both something that derives from a Mus musculus, and something that derives from a bone marrow. A 4470 is a cell line.

Ara-C-resistant murine leukemia cell line	SubClassOf: ’cell line’ has subclass b117h* has subclass b140h* derives from some ’Mus musculus’	An Ara-C-resistant murine leukemia is a cell line. B117Hs, and B140Hs are Ara-C-resistant murine leukemias. An Ara-C-resistant murine leukemia derives from a Mus musculus.

genetic disorder	SubClassOf: disease disjoint(normal, uninfected)	A genetic disorder is a disease. No genetic disorder is any of the following: a normal or an uninfected.

Example of natural language definitions generated from corresponding OWL axioms for the second iteration (Survey 2). *Note: these subclass relations are placed on the subclasses but we illustrate them here for context.

### Survey 1

An interesting outcome of the first survey was that the new natural language definitions exposed an oddity in one of the EFO classes that had not been previously identified. The definition for ‘Ara-C-resistant murine leukemia’ indicated that the subclasses ‘b117h’ and ‘b140h’ were both types of this class, implying that they were diseases rather than cell lines. Ontologically, the classes are subtypes of cell line; however, it is clear that the label for this class is incorrect and would be better served by, for example, appending ‘cell line’ to the end of the class label. The transformation into natural language may have made this anomaly more visible, although it is also possible that the basic ‘crowd sourcing’ of having many people look at definitions, either logical or textual, would bring the same benefit—this will be an avenue for future study.

Many comments on the generated language concerned the nature of the generated nouns. For example, the annotation label *HeLa* was turned into the lexical entry ‘he la’, *HomoSapiens* into ‘homo sapiens’, and *BCell* into ‘b cell’. All these are incorrect, abusing domain conventions of capitalisation and word boundaries. This was in reality an oversight, applying to labels a word division method (based on capitalisation) that is appropriate only for identifiers like *#PartOf*. This highlights the importance of paying attention to conventions of domain language, and was corrected in the program that generated the materials for Survey 2.

The first survey results also revealed an interesting trend towards a desire for simplicity in definitions. The class definition that was deemed most understandable was *BDCM* (described in Table 3), which only asserts that the class is a cell line. The most common remark otherwise was for class *GM18507* (also described in Table 3). Here, participants commented that the line ‘has as quality a male’ was confusing. Similarly, some comments were also made on the language of ‘bearer of’ in the context of a disease; such relationships come from using the relation ontology [30] as part of the OBO process.

Overall in Survey 1, the modal answer given was the 2nd highest rank (a score of 4—see Table 2), which appears to indicate, in this limited response, that answers were at least some way to conveying an understandable meaning.

### Survey 2

As well as dealing more appropriately with labels, the updated NLG program used in Survey 2 had wider coverage and could accept larger ontologies; we were therefore able to verbalise (in five minutes) the whole of the EFO ontology, whereas the earlier version ran only on a selected subset of entities. As a result, the materials for the second survey were subtly different from Survey 1 (though certainly related), so that a direct pairwise comparison is not possible.

In Survey 2 (see Table 4, there were 14 completed sets of answers. All bar two of the participants gave scores of 4 or 5. One respondant provided only comments, largely on the ontological nature of the OWL axioms, rather than a judgement on whether the English was a rendering of the OWL (whether or not it was a sensible axiom). For example, the axiom *bearer_of some ‘breast carcinoma’* which gives the generated English ‘A breast cancer cell line is defined as something that is bearer of a breast carcinoma.’ received the response ‘so my mother is a breast cancer cell line?’. One other respondant gave scores of 1–3, but no comments. Table 4 includes all responses and even when including these two outliers, the responses appear to have shown an improvement on the first survey.

The results from the first part of the second survey suggested that the simple improvements made to the NLG tool had removed some of the prior issues raised, although other difficulties still remained:

• There were fewer comments about naming convention translation errors, such as ’HeLa’ into ’he la’ and the latin ’Homo sapiens’ into ’homo sapien’ as seen in the first iteration. This suggests that domain nomenclature is important to the domain experts when considering ontology definitions. Variations or a loss of precision in these well-accepted naming conventions are clearly unacceptable to users.

• Repetition of the word ‘something’ was seen to be clumsy by many respondants (for examples see Table 5). For the cell line examples, instead of a Hela is something that…’ we could generate ‘A HeLa is a cell line that …’ (where the *genus* replaces the ‘something’). There would still be repetition, but it would be of a more relevant word. Some more complex grammatical structures might also be used, but possibly at a cost of ‘easy reading’. Again, future work will test more variations of sentence forms. For instance, the rules used in these surveys generate ‘A HeLa is something that derives from a cervix and something that derives from an epithelial cell …’, because the rule for expressing an aggregated list of classes assumed they would be put into a large noun-phrase. This has the advantage of working in all cases, but can give clumsy results. In the latest version we have now added a rule that tries a verb-phrase first, to obtain ‘A HeLa derives from a cervix and derives from an epithelial cell…’, before using the previous rule that will work in all cases.

• Similarly, the use of ‘ontology language’ such as formal relationship labels from the relations ontology [30] was unattractive to some participants. There were many comments on questions in which the use of words such as ‘quality’ and ‘disposition’ in the English definition was disliked, as they did not fit with conventional domain language. This may also suggest that domain terminology needs to be accounted for in such an exercise and that alternative, domain-friendly labels would be one useful addition for ontologies that use such language.

• There were also suggestions that the ordering of sentences could be improved and that alternative wording for premodifiers would improve the definitions. Some of these issues are being addressed in our current research, such as experimenting with grouping axioms under ordered sub-headings (as suggested by [31] in an analysis of encyclopedia entries).

In the second part of this survey, exploring alternative wordings for axioms containing some of the properties (see Figure 3), the results suggested an interesting overall pattern: that the definition that was the most natural to read was almost always different from the definition that most captured the meaning of the OWL axioms. Arguably this is to be expected, since the definitions are being evaluated here for different purposes, but this also suggests that there is a trade-off between fluent, readable English and semantic precision. Definitions that simply mirror the OWL as closely as possible are potentially not desirable to the user, although definitions that result in a loss of precision in terms of nomenclature are also not desirable.

In three questions (Q13-Q15 in Figure 3), we explored various combinations of aggregation and elision:

• Q13 contrasts a non-aggregated form (Q13a, one sentence per axiom) with three aggregated forms of decreasing prolixity. Interestingly, one of the aggregated forms (Q13b) was judged more faithful to OWL than the non-aggregated form. As expected, the most concise aggregated form (Q13d) was judged most natural to read, although less faithful to OWL. One of the aggregated forms (Q13c) had a serious structural ambiguity which participants apparently detected, since they all rejected it.

• Q14 offered the generated definition (Q14a) along with two more concise versions, one of which (Q14c) used elision *within the class names* (abbreviating ‘a medial geniculate nucleus or a lateral geniculate nucleus’ to ‘a medial or lateral geniculate nucleus’). This version (Q14c) was the clear winner for naturalness, although rated less faithful to OWL.

• Q15 explored a similar within-term elision for a longer list, by offering an alternative (Q15b) in which ‘leukemia’ was removed from all subclasses. This was a somewhat different case from Q14c since the list was longer, and the elided noun ‘leukemia’ was not attached to the last member of the list, and the outcome was also different, with the elided form dispreferred on both counts (naturalness and faithfulness).

These findings are only indicative since the data are sparse, but they suggest several avenues to explore. As expected, we find a trade-off between naturalness and faithfulness, but we cannot assume that in all cases the direct non-aggregated verbalisation will be judged most faithful. Again, as expected, versions that have been streamlined by elision are usually judged more natural, although less faithful, but this seems to depend partly on how skillfully the elision is applied: the judgements and comments suggest that appropriate elisions can preserve faithfulness, and that clumsy or ambiguous elisions can damage naturalness.

## Conclusions

We have presented a prototype for the specific NLG task of generating text definitions from logical descriptions of classes. We verbalised a selection of classes from the OWL axioms in EFO and undertook two informal surveys. Whilst it is not possible to draw statistically significant conclusions from these kinds of survey, they have suggested that the text definitions we generated are understandable and useful within the context of an ontology with sparse use of text definitions.

Suggestions for improvements in the English realization of the definitions have been gathered and some have been acted upon. Our initial verbalisations made the OWL semantics explicit (for example, by saying ’Every cell line is …’). This was found to be obstructive to understanding and we replaced it with a simple ’A cell line is…’ formulation. Similarly, explicit verbalisations of all relationships was seen to reduce understanding; for example, qualities of cells. Such dependent entities could, when the entity forms part of another sentence, become adjectival forms of the independent entities in which they inhere (‘cell that has quality female’ becomes ‘female cell’). There are other, simpler forms, of such axioms—‘cell x is female’. Similarly, the formal ontological nature of some relationships reduced understanding; this suggested that alternative wording be found that is closer to the user’s domain without loss of precision. Our second verbalisation only made small changes to the generated English, mainly with respect to the proper use of labels. This appears to have had a positive effect, suggesting the importance of staying as faithful to domain conventions of nomenclature as possible. The results of our second survey suggests that the generated English definitions were found to be a satisfactory way of determining the meaning of the OWL axioms. The results of the two surveys are not directly comparable as the style of evaluation and questions asked were not the same in each survey. However, it was noticeable that some of the criticisms in the first survey were not repeated in the second, suggesting some of these earlier problems had been resolved.

Our second survey explored some options for removing ‘ontological complexity’ by changing the lexicalisation of the property form. These test versions of sentences were apparently found more pleasing, but perhaps at the cost of ontological precision; results suggesting that when a definition is most easy to read as English it does not capture the OWL definition in quite as much detail as an alternative, less readable definition.

A more thorough exploration of all aspects of these renderings is necessary. There is, however, a suggestion that a variety of output styles is possible and needed, with some being closer to domain language, some making more of OWL’s semantics explicit whilst others preserve more of the ontology’s form. It would appear that there is not one form of output that will satisfy all types of user. In the short term we will continue to generate EFO text definitions and improve their quality for that user group. It would appear that generated English that is faithful to both the OWL semantics of axioms and the full ontological nature of an axiom, whilst remaining readable, is far from easy. Overall, however, a systematic survey of appropriate verbalisations of definitions is required to inform such renderings.

The main contribution of this work is the application of a variety of linguistic NLG techniques to produce coherent paragraphs of text for generating natural language style definitions for ontologies authored in OWL. This specific task is a subset of the wider OWL verbalisation task that can be of immediate use to ontology developers. Our approach requires no intervention and can be applied to any domain to produce natural language definitions of reasonable quality. Whilst there remains much to do to improve our verbalisations, we are encouraged by the reactions to these early attempts. Based on the reaction from our Survey, the providers of EFO are now including these generated text definitions in their latest release (version 2.10). We foresee that generic tools for verbalisation of ontologies from logical descriptions will be both possible and useful to a wide variety of users.

## Materials and method

### Description generator

The description generator accepts as input an ontology encoded in OWL/RDF, and produces as output a text file that lists the atomic entities, in alphabetical order of their English names, accompanied by descriptions in English sentences. The descriptions will contain inferred statements only if these are already included in the input file; they are not added by the program itself. For our specific task of producing verbalisations of natural language definitions, the reasoner only supplies any inferred subsumption relationships; the definitions only require access to the statements that differentiate the class in question from its superclasses and the asserted statements plus the complete subsumption hierarchy are sufficient for this task; access to ‘everything that is known’ about a class is not necessary. The completion of the subsumption hierarchy is something that we could do as part of the on-line tool, via the OWL API [32], but the costs in an on-line setting can be high. This is, however, something would be better added to a tool such as a plugin to Protégé.

To produce the descriptions, the program collects all the axioms relating to a given entity, groups them according to common structure, realises each group through an English sentence, and assembles the resulting sentences into a paragraph. Sentence generation is accomplished using a generic grammar based on logical patterns in OWL, together with a lexicon for realising atomic entities. A provisional lexicon is derived automatically from the identifier names or annotation labels in the input ontology; if desired it can be improved by hand.

This version of the system has several limitations:

• First, its coverage is a slightly restricted subset of OWL 2 that excludes inverse properties, enumerated classes (OneOf) with multiple arguments and some datatypes; the output therefore occasionally includes a formula in OWL Functional Syntax rather than English, indicating that the axiom uses an OWL functor that is not covered.

• Second, it is implemented in SWI Prolog, a language that is highly suitable for language processing and well-suited to fast prototyping [33], but somewhat under-performant. For small ontologies (e.g., the well-known training examples People+Pets and PizzaTopping) the response is almost instantaneous, but larger ontologies may take several minutes (e.g., 5 minutes for the whole of EFO). When all the rules for verbalisation are settled and tested, a Java implementation will be developed.

• Third, the methods used for deriving lexical entries from identifiers and labels are rudimentary (and of course they assume that these names are based on English).

• Finally, the grammar for realising logical patterns is mostly based on intuition (either our own, or that of previous researchers—see above); as yet there are no systematic empirical studies on the best linguistic formulations.

The process of generating descriptions has five phases:

1. Transcoding from OWL to Prolog.

2. Constructing a lexicon for atomic entities.

3. Selecting the axioms relevant for describing each class.

4. Aggregating axioms with a similar structure.

5. Generating sentences from (possibly aggregated) axioms.

**Figure 4:**
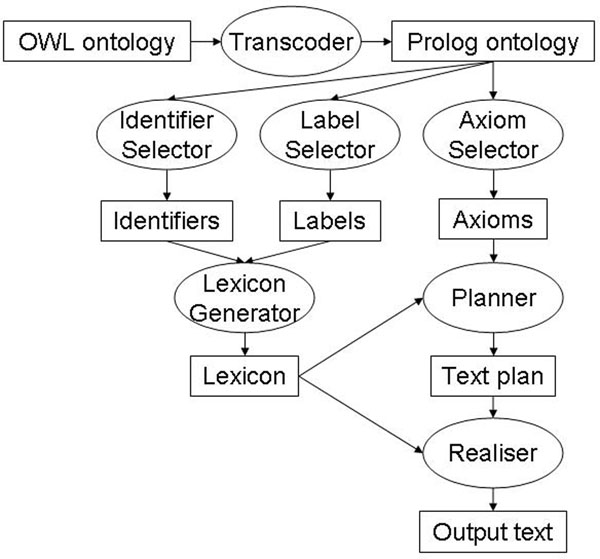
**The architecture of the OWL verbaliser**. Architecture of the natural language definition generator.

The architecture of the system (somewhat simplified) is shown in Figure 4. In this diagram, rectangles denote data files, including the input (an OWL file) and the output (a text file), as well as various Prolog files computed along the way (e.g., the lexicon). Ovals represent processes, usually implemented as Prolog modules. The application is now available as a web service [34]. In addition to obtaining the class description text, users can request alternative text outputs such as a straight axiom-to-sentence verbalisation with no grouping, and can view some of the intermediate files (axioms, lexicon) and some results from analytical programs (e.g., frequency analysis of axiom patterns).

#### Transcoding to Prolog

This stage covers the processes called ‘Transcoder’, ‘Identifier selector’ and ‘Label selector’ in Figure 4. The input is a file such as *efo.owl* in OWL/RDF format. In the first step, we convert to a convenient Prolog format, in which each axiom is encoded by a single Prolog term, and identifiers are standardised by replacing abbreviated IRIs by complete ones. During this process, annotation assertions including labels for identifiers are stored for future reference in Prolog form, and for convenience all atomic terms are also listed through their full identifiers (this is useful when compiling the lexicon, and also when planning the output document that is akin to a technical dictionary or encyclopedia). The format used in these files is shown below through six representative terms from the EFO ontology (the actual files contain over 3000 Prolog terms each).

With this information separately stored, the main part of the ontology is converted to a file in which each Prolog term encodes an axiom, and non-axiomatic statements such as prefixes, annotations and declarations are excluded.

Technically, the conversion is performed using freely available software: the Manchester OWL API that transforms from OWL/RDF to OWL/XML, and the Prolog library for transforming any XML file into a list of Prolog terms. The Prolog form that we use for an axiom is almost exactly the same as OWL Functional Syntax, the only differences being that arguments are separated by commas, not by spaces, and functors begin with a lower-case letter; for examples see below.

#### Constructing a lexicon

In this stage, labelled ‘Lexicon Generator’ in Figure 4, a provisional lexical entry is computed for each entity identifier. To do this, the program first checks whether a label is provided in an annotation assertion; if so, the lexical entry is based on this label, otherwise it is based on the identifier itself. To obtain the lexical entry from an identifier, the program discards the namespace, then splits the remaining string into words on the assumption that word boundaries are indicated by underline characters or capital letters; some simple heuristics are then applied to massage the resulting word string into a plausible English phrase. It is assumed that the syntax of each phrase will be severely constrained as follows: individuals are expressed by proper names; classes by common nouns (with singular and plural forms); and properties by transitive verbs (simple or compound) with slots for a subject and an object. Lexical entries are saved as Prolog terms with four arguments: identifier, part of speech, singular form, and plural form (if relevant).

As can be seen, the lexicon is reliant on the names/labels provided by the ontology builder, and uses no other source of evidence. The treatment of identifiers (minus namespace) and labels is slightly different, on the assumption that capital letters will be used often as word boundaries in identifiers, but not in labels. Thus on retrieving the string ‘partOf’ from an identifier, we interpret the capital letter ‘O’ as a separator and segment into ‘part of’; applied without any refinement, this rule would mean that for example ‘HeLa’ is segmented into ‘he la’ — obviously a bad guess since to the educated eye ‘HeLa’ looks like a single technical term. However, since labels (unlike identifiers) can include spaces, the use of capital letters as separators is rare, and accordingly it is best to assume that a connected string like ‘HeLa’ should be left as it is. We therefore segment labels on the principle that the only word separators are spaces and underlines. Having derived a word string from the identifier or label, it remains to apply some simple transformations in order to obtain a lexical entry of the appropriate kind. At present, as already emphasized, these are very rudimentary, and we plan to replace them by rules based on part-of-speech analysis. The strings in most need of revision are those expressing properties — for instance, ’has part’ or ’part of’, which would be transformed as follows:

• For any string of the form *has X*, make a singular verb phrase *has as X* and a plural *have as Xs*; thus we obtain ‘has as part’ and ‘have as parts’.

• For any string of the form *X of*, where X is not a verb in the present tense (does not end in -s), make a singular verb phrase *is X of* and a plural *are Xs of*; thus we obtain ‘is part of’ and ‘are parts of’.

#### Selecting axioms for each entry

This and the following stage belong to the process labelled ‘Planner’ in Figure 4. Once the lexicon has been built, the ontology is searched for axioms that describe each class, property and individual in the lexicon (i.e., each atomic entity). For example, to describe the atomic class *EFO_0002095* the algorithm retrieves all axioms in which this class occurs as a top-level argument (e.g., A or B if the axiom is subClassOf(A,B)) obtaining the following set:

The algorithm carries out the same search for every lexical entry, so that each atomic entity is associated with a subset of relevant axioms. The grouping of axioms within each subset occurs in the next stage.

#### Aggregating similar axioms

To complete the text plan, the axioms selected as relevant for a given entity are grouped by similarity, so that they can be realised more concisely in aggregated sentences. As an alternative we could simply generate a sentence for each axiom, but the resulting text would contain many repetitions; for example, for the set of axioms for cell line *22rv1* we would obtain:

A 22rv1 is a cell line.

A 22rv1 is bearer of a prostate carcinoma.

A 22rv1 derives from a Homo sapiens.

A 22rv1 derives from a prostate.

To obtain more fluent descriptions, our algorithm combines axioms that share a common pattern and differ in only one constituent. Thus in the example we are considering, it finds three axioms having the following abstract form:

These are combined to obtain the following aggregated axiom in which the varying constituent is replaced by a list:

The grammar can then realise the aggregated axiom by a single sentence rather than several sentences. For more details, see [28].

#### Generating sentences

The final stage corresponds to the process labelled ‘Realiser’ in Figure 4. For each entity to be described, the text plan specifies a set of (possibly aggregated) axioms; it remains to generate a sentence for each axiom (or aggregated axiom), thus obtaining a description of the class (or other atomic entity). This is done by feeding each axiom to a Definite Clause Grammar (formalism for expressing a context-free phrase-structure grammar in a logic programming language such as Prolog; see [33, chap. 4]), with rules for (nearly) every logical pattern in OWL-DL; this grammar will consult the lexicon whenever it needs to express an atomic entity. As an example, here is the rule used for realising a two-argument statement with the functor *equivalentClasses*; as can be seen, it presupposes a further rule for realising classes by indefinite noun phrases:

Translated into English, this means that if you want to express a logical pattern of the form *equivalentClasses*(*C*,*D*), construct a sentence in which the first constituent (i.e., the subject) is a noun phrase expressing class *C* using the indefinite article, the next three constituents are the words ‘is defined as’, and the final constituent is a noun phrase expressing class *D* (again using the indefinite article). At present we have no heuristics for ordering axioms within a description, so the sentences are assembled into a paragraph following the same order in which the axioms were originally retrieved from the ontology. The final output is a text file organised on the lines of a glossary, listing the classes and other atomic terms in alphabetical order of their English names, each accompanied by a paragraph of description. For an excerpt from the output for the EFO ontology, see Table 3.

### Evaluation studies

We used a simple evaluation strategy of generating textual definitions from EFO and then showing them to potential EFO users for comment. We did two passes at this evaluation. From the observations made during the first pass, changes were made to the generation program that produced the definitions; these were then presented in the second pass.

#### Materials

##### Survey 1

In the first pass we verbalised a subset of 50 cell lines from EFO. We used cell lines as they represent a substantial portion of EFO; the topic is broadly accessible to the target audience and this portion of EFO lacks definitions. These included 45 without (and 5 with) hand-crafted text definitions; 5 also had necessary and sufficient conditions while 45 had only necessary conditions from just a subclass axiom to several restrictions. This covered a range of common encoding paradigms in OWL—a collection of restrictions upon another kind of entity, some only necessary conditions and some that were both necessary and sufficient. This subset used some of the more common properties used in EFO. The cells covered a range of human and mouse cells, some of which exhibited diseases. Table 3 provides some examples of text definitions; the supplementary information contains the whole set of generated definitions used in this evaluation.

We used the output from 10 of these in a simple survey and asked participants to what extent they thought the definitions were readable so that their intention could be understood. Participants were also able to add specific comments to each definition.

##### Survey 2

The generated texts for the second survey were produced by an updated version of the NLG program, with the following changes:

• The annotation label was processed differently from the URI fragment for the lexical entry, so that technical terms like ‘HeLa’ were no longer subdivided inappropriately into ‘he la’.

• The re-implemented description generator was able to perform over the whole of EFO (as memory constraints were improved) so definitions in some of the questions included an example for a disease and for an anatomical part (as well as cell lines).

• The grammar of the program was substantially extended to cover not only ***EL***++ but most of OWL-2.

We divided this second survey into two parts. In the first part we selected 10 generated descriptions, as before, to cover a wide range of property types and wordings, and asked the participants whether the generated English accurately captured the meaning of the OWL axioms. In the second part we tested (i) a variety of alternative forms of English for some of the properties used in EFO and (ii) variations with and without aggregation, and various degrees of elision of repeated noun phrases and ‘something that’ phrases.

An example of (i), variations in the English rendering of properties, is the axiom *bearer_of some ’cervical carcinoma’* in which we tried the following hand-crafted variations for ‘bearer_of’:

• something that is bearer of a cervical carcinoma

• something that bears cervical carcinoma

• something that carries cervical carcinoma

• something that is cervical carcinomic

• something that has cervical carcinoma

We created these sentences by varying how the property was rendered, from a straightforward mapping to natural language to forms that were judged to be progressively ‘easier’ English, so that ‘bearer of’ becomes ‘bears’ and then ‘carries’ and finally ‘has’ (cervical carcinoma). Taking a similar approach, ‘cell has quality female’ becomes ‘cell is female’ and so on. Second, we adjusted the form of the filler of the property, so in one case ‘carcinoma’ became ‘carcinomic’, to put it into some kind of adjectival form.

For (ii), versions with and without aggregation were generated automatically, but versions eliding ‘something that’ and noun phrases such as ‘geniculate nucleus’ were handcrafted, for example:

• **No elipsis:** A geniculate nucleus is defined as something that is a medial geniculate nucleus, or is a lateral geniculate nucleus.

• **Elipsis of ‘something that’ and ‘is’:** A geniculate nucleus is defined as a medial geniculate nucleus or a lateral geniculate nucleus.

• **Elipsis of ‘geniculate nucleus’:** A geniculate nucleus is defined as a medial or a lateral geniculate nucleus.

For these alternative definitions, we wanted to gain insight into which of these definitions the participants thought were closest to natural language and therefore easiest to read and also which of the definitions participants thought were closest to capturing the meaning of the OWL. The full set of questions can be seen in Figure 3.

#### Procedure

In order to evaluate the verbalisations from the two iterations, two on-line surveys were created (see supplementary information).

##### Survey 1

In the first survey, a sample of 10 of the 50 verbalisations was selected based on the widest range of axioms (i.e. number and type on each class). Participants were asked to rate on a scale of 1 to 5 how much they thought the definitions were readable such that their intention could be understood. Participants were also able to add specific comments to each definition.

##### Survey 2

The second survey was split into two parts. In the first part, a sample of 10 definitions was again selected based on the widest range of axioms (i.e. number and type on each class). This included definitions with equivalent conditions, necessary conditions and disjoints. The sample also contained definitions for parts of the ontology other than just cell lines, since the second pass of the natural language generator went across all classes. For this reason, three of the examples were selected from subclasses of anatomy and disease. The participants were asked to evaluate how well they thought the meaning of the OWL axiom shown was described by the text.

In the second part of the survey, we designed several alternative natural language definitions for five OWL axioms, again selecting five fairly different sets of axioms. We asked the participants to rank which of the alternative definitions they thought (i) was most natural to read, and (ii) captured the meaning of the OWL most accurately. Since the survey required a knowledge of OWL, the invited participants were limited to those from the OWL ontology community. Questions did not mandatorily require an answer and an optional comment could be made on each question.

## Competing interests

The authors declare that they have no competing interests.

## Authors’ contributions

All authors contributed to this article. RP and SW designed and implemented the NLG system. AT implemented the NLG tools website. JM and RS created the study. All authors had input on the form of the language generated.
